# Trajectories of cardiovascular disease risk and their association with the incidence of cardiovascular events over 18 years of follow-up: The Tehran Lipid and Glucose study

**DOI:** 10.1186/s12967-021-02984-2

**Published:** 2021-07-16

**Authors:** Fatemeh Koohi, Nooshin Ahmadi, Farzad Hadaegh, Siavash Safiee, Fereidoun Azizi, Davood Khalili

**Affiliations:** 1 Department of Epidemiology, School of Public Health and Safety, Shahid Beheshti University of Medical Sciences, Tehran, Iran; 2 Department of Epidemiology and Biostatistics, Research Institute for Endocrine Sciences, Shahid Beheshti University of Medical Sciences, Tehran, Iran; 3 Prevention of Metabolic Disorders Research Center, Research Institute for Endocrine Sciences, Shahid Beheshti University of Medical Sciences, Tehran, Iran; 4 Faculty of Medicin, Tehran Medical Branch, Islamic Azad University, Tehran, Iran; 5 Endocrine Research Center, Research Institute for Endocrine Sciences, Shahid Beheshti University of Medical Sciences, Tehran, Iran

**Keywords:** Cardiovascular risk score, Risk prediction, Trajectory analysis, Cohort study

## Abstract

**Background:**

Understanding long-term patterns (trajectories) of cardiovascular diseases (CVD) risk and identifying different sub-groups with the same underlying risk patterns could help facilitate targeted cardiovascular prevention programs.

**Methods:**

A total of 3699 participants of the Tehran Lipid and Glucose Study (TLGS) (43% men, mean age = 53.2 years), free of CVD at baseline in 1999–2001 and attending at least one re-examination cycle between the second (2002–2005) and fourth cycles (2009–2011) were included. We examined trajectories of CVD risk, based on the ACC/AHA pooled cohort equation, over ten years and subsequent risks of incident CVD during eight years later. We estimated trajectories of CVD risk using group-based trajectory modeling. The prospective association of identified trajectories with CVD was examined using Cox proportional hazard model.

**Results:**

Three distinct trajectories were identified (low-low, medium-medium, and high-high risk). The high-high and medium-medium CVD risk trajectories had an increasing trend of risk during the time; still, this rising trend was disappeared after removing the effect of increasing age. Upon a median 8.4 years follow-up, 146 CVD events occurred. After adjusting for age, the medium-medium and high-high trajectories had a 2.4-fold (95% CI 1.46–3.97) and 3.46-fold (95% CI 1.56–7.70) risk of CVD compared with the low-low group, respectively. In all trajectory groups, unfavorable increasing in fasting glucose, but favorable raising in HDL and decreasing smoking and total cholesterol happened over time.

**Conclusions:**

Although the risk trajectories were stable during the time, different risk factors varied differently in each trajectory. These findings emphasize the importance of attention to each risk factor separately and implementing preventive strategies that optimize CVD risk factors besides the CVD risk.

**Supplementary Information:**

The online version contains supplementary material available at 10.1186/s12967-021-02984-2.

## Introduction

Cardiovascular disease (CVD) remains the leading cause of death globally, and over 75% of these occur in low- and middle-income countries; moreover, it is a significant barrier to human development [1]. In 2016, an estimation of 17.9 million people died from CVDs, responsible for 31% of all deaths [1]. In Iran, CVD is the most common cause of death and responsible for 46% of all deaths and 20%-23% of disease burden [2].

Although CVDs are multifactorial diseases, their morbidity and mortality can be reduced by managing and controlling their modifiable risk factors. The INTERHEART study showed that the underlying risk factors for CVD are similar globally. More than 90% of the risk for incident myocardial infarction is attributable to nine modifiable risk factors: abdominal obesity, diabetes mellitus, hypertension, unhealthy diet, abnormal lipids, lack of regular physical activity, smoking, alcohol consumption, and stress [3]. These factors have a constant and progressive impact on total CVD risk, so evaluating all known modifiable risk factors to provide a detailed absolute CVD risk is recommended to prevent CVD appropriately and cost-effectively [4].

Using cardiovascular risk scoring in the guidelines has a long history. The American College of Cardiology (ACC) and the American Heart Association (AHA) released a new approach on the assessment of cardiovascular risk, in which they developed the race- and sex-specific Pooled Cohort Equations to predict the 10-year risk of a first hard CVD event in ages 40 to 79 years [5]. However, information on the effectiveness of these risk scores in the primary prevention of CVD and the estimated risk changes over time is limited. Several studies reported reducing predicted global CVD risk after using these risk scores [6–8]. Some other studies revealed little evidence for the effectiveness of risk assessment on change in predicted CVD risk and CVD risk factors [9–11]. A previous study also suggested that repeating risk assessments within four years can modestly improve prediction compared with a single risk assessment [12].

Understanding how variations in CVD risk scores during adulthood contribute to the risk later in life could help to facilitate targeted cardiovascular prevention programs [13]. However, there is limited information on identifying long-term patterns of CVD risk scores (referred to as trajectories) and then linking these patterns to the incidence of CVD events. Here, first, we identified the trajectories of the ACC/AHA pooled cohort CVD risk score using four assessments over ten years. Then we examined the association of these trajectories with the incidence of hard CVD events, including non-fatal myocardial infarction, fatal coronary heart disease, and fatal or non-fatal stroke during the following years.

## Methods

### Study population

Tehran Lipid and Glucose Study (TLGS) is a prospective population-based cohort study. In 1999–2002 the first phase of the study recruited 15,005 men and women aged three years and more, living in Tehran, district No.13, a representative sample of an urban Iranian population. The participants of TLGS have been followed up for 20 years, approximately every three years, and so far, the data have been collected across six subsequent phases. Details regarding the methods and design of TLGS have been reported previously [13–15]. In brief, trained social workers invited participants to the TLGS unit and took written informed consent from them. Demographic and lifestyle information was obtained using self-reported standard questionnaires. Then trained physicians interviewed participants to get past medical history, smoking habits, and physical exam. Systolic and diastolic blood pressure (mm Hg) was taken as the average of two measurements in the sitting position after five-minute rest using a standard mercury sphygmomanometer. Anthropometric measurements were taken according to the standard protocols with shoes removed and the participants wearing light clothing. A blood sample is drawn from all study participants after a 12–14 h overnight fast. A second blood sample is taken two hours after glucose ingestion according to the standard protocol. Biochemical measurements including fasting plasma glucose (FPG), two-hour post-glucose load (2-hPG), and all blood lipid analyses were performed at the TLGS research laboratory on the day of blood collection using Selectra 2auto-analyzer (Vital Scientific, Spankeren, Netherlands). Diabetes was defined by fasting glucose ≥ 126 mg/dL or the use of diabetes medication. The Ethics Committee of the Research Institute for Endocrine Sciences, Shahid Beheshti University of Medical Sciences, approved all protocols of TLGS.

For the current study, all participants of TLGS aged 40–79 years who attended the baseline assessment and at least one additional follow-up re-examination between the second (2002–2005) and fourth cycles (2009–2011) were included (n = 4268). After excluding participants with prevalent CVD (n = 331), those with missing data on the CVD risk score at baseline (n = 161), and those with missing data on the CVD risk score during all examination cycles one to four (n = 77), 3699 participants were included in the trajectory analysis. We also followed those attending the fourth examination cycle and did not have prevalent CVD and missing data in this cycle (n = 2619) up to March of 2018. This sub-sample was used in survival analysis to link the CVD risk trajectory groups (defined using the original sample) to incident hard CVD, including non-fatal myocardial infarction, fatal coronary heart disease, and fatal or non-fatal stroke.

### Assessment of cardiovascular risk

The Pooled Risk Equations recommended by ACC/AHA for non-Hispanic white men and women were used to calculate the 10-year risk of hard CVD [5]. These equations included covariates of age, total cholesterol, HDL cholesterol, treated or untreated systolic blood pressure, history of diabetes (Y/N), and current smoking status (Y/N); the validity of these equations was previously evaluated in the TLGS [16]. The risk score components were drawn from questionnaires and clinical examination data at six examination cycles: 1999–2001, 2002–2005, 2006–2008, 2009–2011, 2012–2014, and 2015–2018.

### Outcomes

The primary outcome for the prospective analysis was hard CVDs, including non-fatal myocardial infarction, fatal coronary heart disease, and fatal or non-fatal stroke [5, 16]. The TLGS participants are followed up for any medical event, including CVDs and death during the previous year, by telephone calls annually. An outcome committee consisting of an internist, endocrinologist, cardiologist, and epidemiologist adjudicates all events. Deaths are confirmed through death certificate records. The cause of death is determined based on the death certificate and detailed review of medical records and all information provided by attending physicians, medical examiners, and/or family members. For the prospective analysis, participants were followed from the fourth examination cycle (2009–2011) until March 2018.

### Statistical analysis

Data are shown as mean ± standard deviation for continuous variables or as number (%) for categorical variables. To identify distinct baseline to 10-year CVD risk trajectories, we used group-based trajectory models in a Stata plugin program (Stata Proc Traj) [17]. This method models the dependent variable (ACC/AHA risk score) as a function of time. It identifies individuals' clusters following a similar underlying trajectory on the dependent variable over time within a population, based on a maximum likelihood method [17, 18]. We applied a censored normal model [19] to identify distinct trajectories of the CVD risk score. We fitted different models to determine the "best" model, treating CVD risk score as the dependent variable and time at follow-up as the independent variable. We developed different models by varying numbers of groups, ranging from two to five groups, and shapes (linear, quadratic, and cubic). We then compared them using Bayesian Information Criteria (BIC) and a sufficient proportion of participants in each subgroup [18]. The results of this process are summarized in Additional file 1: Table S1. To ensure the adequacy of the selected model, we assessed four models that fit diagnostic criteria as suggested by Nagin [20]: (1) average posterior probability of assignment for each group j (AvePPj) equal to 0.7 or greater for all groups; (2) the odds of correct classification (OCCj) equal to 5 or higher for all groups; (3) similarity between the proportion of a sample assigned to a specific group and the group probabilities estimated from the model; and (4) narrow CIs of the estimated proportion. Using this approach, we identified three distinct trajectories for CVD risk score. The selected CVD risk trajectory groups were labeled according to their CVD risk at baseline and examination cycle 4 to show the trajectory of the risks during the time. The median of the trajectory groups' risk was 3%, 17%, and 38%, which are compatible with the ACC/AHA risk categories of low < 7.5%, medium ≥ 7.5 to < 20, and high ≥ 20.

After identifying CVD risk score trajectory groups, we evaluated the associations of trajectory subgroup membership (as a categorical exposure) with incident hard CVD after the fourth examination cycle using Cox proportional hazards regression model. Given that total cholesterol, HDL cholesterol, systolic blood pressure, history of diabetes, and current smoking status are included in the ACC/AHA pooled cohort risk algorithm, we did not consider them in the multivariate model. Since age is a strong non-modifiable risk factor and increases during follow-up, it was adjusted in model 2—education level and family history of premature CVD as non-modifiable factors adjusted in model 3. Moreover, BMI, lipid-lowering drug use, and physical activity have not been included in the ACC/AHA pooled cohort model. However, we did not adjust them because their intermediate variables (i.e., blood pressure, cholesterol, and diabetes) are still in the original model. This adjustment results in underestimating HRs for risk trajectories. Statistical significance was considered using a two-sided P < 0.05. All analyses were performed using Stata software version 14 (STATA Corp., TX, US).

### Sensitivity analysis

As a sensitivity analysis, to assess the impact of increasing age on the CVD risk estimates and the overall shape of the trajectories, we repeated trajectory analysis by calculating new risk scores using risk factor values at each examination cycle but the age at the first exam.

Besides, we assessed the trend of each risk factor included in the ACC/AHA risk score containing systolic blood pressure (SBP), total cholesterol, HDL, fasting blood sugar (FBS), smoking, as well as CVD risk scores by the trajectory groups identified in the primary analysis using a generalized estimating equation (GEE) analysis.

## Results

### Participant's characteristics

The trajectory patterns in CVD risk were examined among 3699 participants aged 40–79 during four examinations in 10 years. The mean (SD) age of participants in the original sample was 53.2 (9.3) years, with a higher level of total cholesterol, LDL, triglycerides, and a higher prevalence of smokers than the sub-sample followed for CVD events. Participants in the sub-sample were older, with a higher proportion of lipid-lowering drug and antihypertensive drug and anti-diabetic drug use. These differences between the two samples were because of increasing age. The baseline characteristics of the original and the sub-sample at baseline and examination cycle four, respectively, are shown in Table 1.

**Table 1:** Characteristics of the participants

Characteristics*	Original Sample† (n = 3699)	Prospective sub-Sample‡ (n = 2522)	P⁑
Age, y	53.2 ± 9.3	61.6 ± 8.7	< .0001
Male, n (%)	1588 (42.9)	1036 (41.1)	1.000
Body mass index, kg/m2	28.0 ± 4.5	29.1 ± 4.9	< .0001
Total cholesterol, mg/dL	225.1 ± 46.8	204.4 ± 42.1	< .0001
LDL cholesterol, mg/dL	144.7 ± 38.7	123.9 ± 35.2	< .0001
HDL cholesterol, mg/dL	42.2 ± 10.9	47.9 ± 11.2	< .0001
Triglycerides, mg/dL, median (IQR)*	169 (119–237)	143 (105–196)	< .0001
Lipid-lowering medication, n (%)	199 (5.4)	329 (13.1)	< .0001
Systolic blood pressure, mm Hg	126.7 ± 20.6	127.2 ± 20.3	< .0001
Diastolic blood pressure, mm Hg	80.7 ± 11.2	79.7 ± 11.3	0.0203
Hypertension, n (%)	1262 (34.1)	1079 (43.1)	< .0001
Anti-hypertensive medication, n (%)	434 (11.7)	587 (23.5)	< .0001
Diabetes mellitus, n (%)	488 (13.2)	530 (21.2)	< .0001
Anti-diabetic medication, n (%)	232 (6.3)	341 (13.6)	< .0001
Current smoking, n (%)	464 (12.5)	225 (9.0)	< .0001

HDL indicates high-density lipoprotein; LDL, low-density lipoprotein

^*^Numbers represent mean ± SD for continuous variables except for Triglycerides that represent the median (IQR); numbers and percentages are corresponding to "Yes" for dichotomous variables

^†^Characteristics for the trajectory sample were measured at the baseline (the first examination cycle)

^‡^Characteristics for the prospective sample were measured at the fourth examination cycle (2005–2008)

**⁑**Paired T-test for continuous variables and McNemar test for categorical variables

### Characterization of trajectories of CVD risk score

Three trajectory groups in CVD risk score were identified according to baseline CVD risk and patterns over time. We evaluated the fitness of the model, and the results are presented in Table 2. For all three trajectory groups, the average posterior probability was more than 0.90, far greater than the recommended value of 0.7, indicating that the model assigned individuals to different trajectory groups with little ambiguity. Further, the value for the OCC was greater than 20 for all three groups, which is also greater than the recommendation of 5 as a general guideline for group-based trajectory modeling (GBTM). Finally, the probability of group membership estimated by the model and the proportion assigned to each group using the maximum probability rule are almost identical.

**Table 2:** Model adequacy results

Trajectory group	AvePP	OCC	P	π
Low-Low	0.98	22	0.74	0.74
Medium-Medium	0.95	64	0.21	0.22
High-High	0.97	625	0.045	0.046

AvePP, average posterior probability; OCC, odds of correct classification; p, the actual proportion of subjects assigned to each trajectory group using the maximum probability rule; π, the posterior probability of group membership estimated by the model

**Fig. 1:**
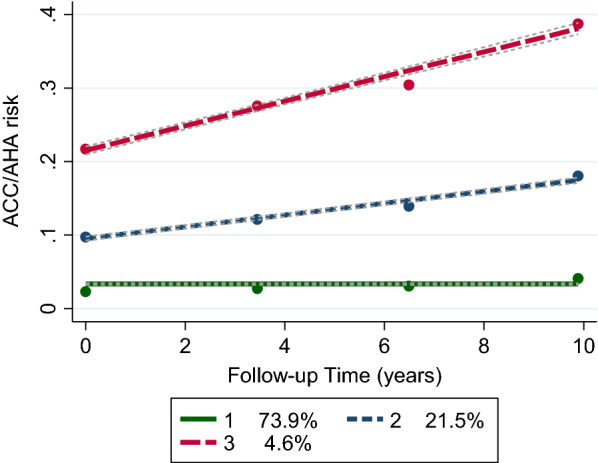
CVD risk score trajectories up to the examination cycle four; circles display the observed values while dotted lines represent fitted trajectories. CVD risk score was modeled as a function of time

Figure 1 depicted the trajectory groups and expected group percentages. We observed that group 1, named low-low, contains individuals with relatively stable CVD risk scores over ten years that the mean risk score ranges from 0.02 to 0.03. However, in group 2, labeled as a medium-medium group, the mean CVD risk score increases from 0.09 to 0.17. Finally, the third trajectory group, named the high-high group, contains individuals whose CVD risk score appears to increase over time with a mean risk score range from 0.20 to 0.38. The two trajectory groups (low–low, 83.3%; medium–high, 16.7%) and four trajectory groups (low–low, 66.4%; low–medium, 20.3%; medium–high, 10.9%; high–high, 2.4%) in CVD risk score are also shown in Additional file 2: Figure S2 and Additional file 3: Figure S2, respectively.

The demographic and clinical characteristics of each of the three groups at baseline and over the follow-up (examination cycle 4) are given in Table 3. Sex distributions were significantly different between the three trajectory groups. For example, the low-low group was mainly composed of female subjects (65.6%), whereas male subjects comprised more than 60% of the other two groups. Further, subjects assigned to the higher groups were significantly older than other groups (p < 0.0001). Clinical variables, including risk factors in the ACC/AHA risk score, were also significantly different between the three trajectory groups. For example, the mean of systolic and diastolic blood pressure in the high-high group was considerably higher than in other groups (p < 0.0001). In addition, total cholesterol was the highest in the medium-medium group baseline and the low-low group at the examination 4. Further, a significantly higher proportion of subjects in the medium-medium group were current smokers than subjects in the other groups.

**Table 3:** Demographic and clinical characteristics by the trajectory groups of CVD risk score

Characteristics*	Low–Low	Medium–Medium	High–High	P†
No. of participants (%)	2740 (73.9)	792 (21.6)	167 (4.6)	–
Men, n (%)	943 (34.4)	517 (65.4)	128 (76.7)	< .0001
Baseline				
Age, mean ± SD, y	49.2 ± 6.5	63.5 ± 5.8	69.1 ± 6.2	< .0001
Body mass index, kg/m2	28.2 ± 4.6	27.5 ± 4.3	27.6 ± 3.8	0.0002
Total cholesterol, mg/dL	223.7 ± 46.7	228.8 ± 45.9	229.8 ± 52.0	0.0110
LDL cholesterol, mg/dL	143.7 ± 38.6	148.4 ± 39.1	145.4 ± 36.5	0.0124
HDL cholesterol, mg/dL	42.6 ± 11.0	41.5 ± 10.8	39.4 ± 9.3	0.0001
Triglycerides, mg/dL, median (IQR)	165 (117–232)	177 (123–245)	179 (133–289)	0.0004
Lipid-lowering medication, n (%)	126 (4.6)	55 (7.0)	18 (10.8)	< .0001
Systolic blood pressure, mm Hg	122.0 ± 17.6	137.3 ± 21.1	152.3 ± 25.9	< .0001
Diastolic blood pressure, mm Hg	80.2 ± 10.7	81.8 ± 12.2	85.4 ± 13.4	< .0001
Hypertension, n (%)	732 (26.7)	406 (51.3)	124 (74.3)	< .0001
Anti-hypertensive medication, n (%)	218 (8.0)	158 (20.0)	58 (34.7)	< .0001
Diabetes mellitus, n (%)	233 (8.5)	173 (21.9)	82 (49.1)	< .0001
Anti-diabetic medication, n (%)	107 (3.9)	86 (10.9)	39 (23.4)	< .0001
Current smoking, n (%)	318 (11.6)	123 (15.6)	23 (13.8)	0.012
CVD risk score, median (IQR)	0.02 (0.01–0.03)	0.09 (0.07–0.12)	0.20 (0.17–0.26)	0.0001
Follow up (Examination cycle four)				
Age, mean ± SD, y	59.0 ± 6.4	73.0 ± 5.8	78.5 ± 5.7	< .0001
Body mass index, kg/m2	29.5 ± 4.9	27.6 ± 4.3	27.6 ± 4.4	< .0001
Total cholesterol, mg/dL	203.8 ± 43.1	196.2 ± 42.5	183.6 ± 40.2	< .0001
LDL cholesterol, mg/dL	122.5 ± 36.2	119.1 ± 37.1	108.6 ± 35.1	0.0005
HDL cholesterol, mg/dL	48.3 ± 11.1	45.8 ± 10.7	42.7 ± 10.4	< .0001
Triglycerides, mg/dL, median (IQR)	144 (105–200)	141 (105–191)	140.5 (103–209)	0.4479
Lipid-lowering medication, n (%)	402 (17.4)	114 (19.3)	24 (23.3)	0.044
Systolic blood pressure, mm Hg	124.8 ± 18.6	137.9 ± 22.0	153.0 ± 24.6	< .0001
Diastolic blood pressure, mm Hg	79.7 ± 11.0	79.6 ± 12.6	82.3 ± 14.1	0.1166
Hypertension, n (%)	952 (41.1)	380 (63.9)	73 (83.0)	< .0001
Anti-hypertensive medication, n (%)	557 (24.1)	232 (39.3)	47 (54.0)	< .0001
Diabetes mellitus, n (%)	437 (18.9)	194 (32.8)	61 (69.3)	< .0001
Anti-diabetic medication, n (%)	274 (11.9)	140 (23.7)	51 (58.0)	< .0001
Current smoking, n (%)	191 (8.2)	59 (10.0)	6 (6.8)	0.370
CVD risk score, median (IQR)	0.03 (0.02–0.06)	0.17 (0.13–0.22)	0.38 (0.31–0.44)	0.0001

*HDL* indicates high-density lipoprotein, *LDL* low-density lipoprotein

^*^Numbers represent mean ± SD or median (IQR) for continuous variables; numbers and percentages are corresponding to "Yes" for dichotomous variables

^**†**^ANOVA or Kruskal–Wallis for continuous variables and chi-square for categorical variables

### Trajectories of CVD risk score and incident hard CVD

During the follow-up period between 2010 and March 2018 (median 8.4 years, range 7–10), 146 incident hard CVD events were identified. The 10-year cumulative incidence of hard CVD was 6.9% based on Kaplan–Meier estimation in the total population. The CVD risk score trajectories were significantly associated with the risk of hard CVD incidence (Table 4). Compared with the low-low trajectory group (group 1), the unadjusted HRs (95% CI) of hard CVD were 4.51 (3.21–6.34) and 8.58 (4.74–15.53) for the medium-medium and high-high trajectory groups, respectively. After adjusting for age at examination cycle four, the medium-medium and high-high trajectory groups had a 2.4-fold and 3.46-fold risk of hard CVD compared with the low-low group, respectively (Table 4, Model 2).

**Table 4:** Associations of CVD risk score trajectories with incident hard CVD (n = 2619)

Events	Events/ n at risk (%)	Model 1*		Model 2†		Model 3‡	
		HR (95% CI)	p-value	HR (95% CI)	p-value	HR (95% CI)	p-value
Low-Low	70/2004 (3.5)	1.00 (Reference)	-	1.00 (Reference)	–	1.00 (Reference)	–
Medium-Medium	63/458 (13.8)	4.51 (3.21–6.34)	< 0.001	2.40 (1.46–3.97)	0.001	2.14 (1.19–3.85)	0.011
High-High	13/60 (21.7)	8.58 (4.74–15.53)	< 0.001	3.46 (1.56–7.70)	0.002	3.14 (1.15–8.52)	0.025

*CVD* cardiovascular disease, *HR* hazard ratio, *CI* confidence interval

^*^Unadjusted model

^**†**^ Model 2 was adjusted for age at examination cycle four

^**‡**^ Model 3 was adjusted for age, education, and family history CVD at examination cycle four

### Sensitivity analyses

**Fig. 2:**
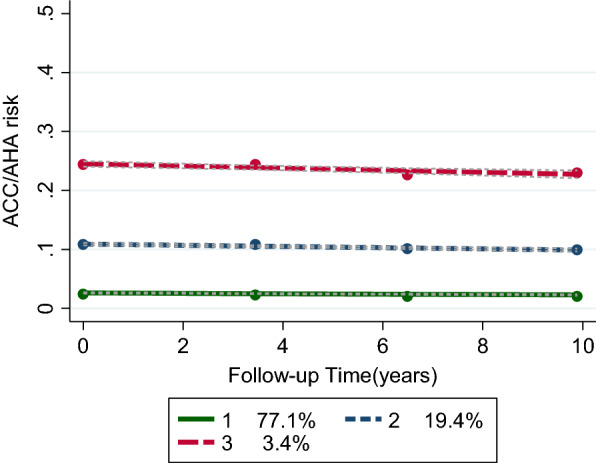
CVD risk score trajectories calculated by keeping age at baseline constant up to the examination cycle four; circles displaying the observed values while dotted lines represent fitted trajectories. CVD risk score was modeled as a function of time

Results from sensitivity analyses examining the trajectory of the risk score calculated by keeping age constant at baseline indicated the same CVD risk trajectories. Still, the increasing risk in high-high and medium-medium trajectory groups disappeared (Fig. 2).

**Fig. 3:**
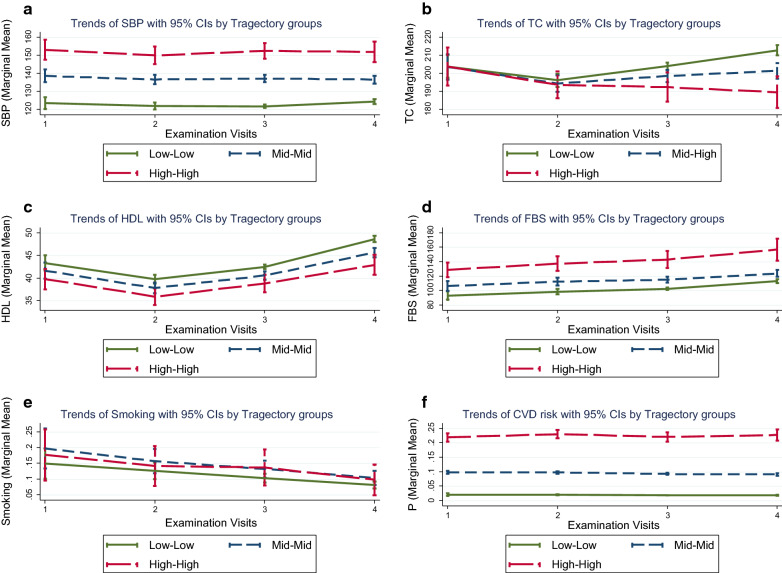
The trend of the risk factors by the trajectory groups depicted in Fig. 1 and Table 3; **A** displays trends of the systolic blood pressure (SBP). **B** shows trends of total cholesterol (TC). **C** displays trends of fasting blood sugar (FBS). **D** reveals trends of the current smoking. **E** indicates trends of the HDL-C. Finally, **F** shows trends of the CVD risk score calculated by keeping age constant at baseline

Additionally, we assessed the trend of the risk factors by each trajectory group (Fig. 3). The results figured out that the systolic blood pressure was relatively stable in the high-high and medium-medium groups and slightly increasing in the low-low group from baseline to examination cycle four. Furthermore, the total cholesterol level was initially decreasing in all trajectory groups. Still, it increased in the low-low and medium-medium groups after the second examination cycle over the follow-up. The concentration of the HDL-cholesterol was growing in all groups, with the highest levels in the low-low group. Besides, the FBS level increased in all trajectory groups while the proportion of the current smoking decreased, with the highest proportion in the medium-medium group. The trend of CVD risk calculated by keeping age constant at baseline was relatively stable in all trajectory groups over the follow-up period.

## Discussion

Our study provides a novel description of CVD risk's longitudinal patterns and their association with CVD events among Iranian adults participating in a prospective population-based cohort study. We used a group-based trajectory modeling approach to identify distinct latent growth trajectories of CVD risk, which are assumed to exist in the population. Such an approach allows us to depict specific groups of individuals who display unique CVD risk patterns over time [20]. Using longitudinal data across four examination cycles, we identified three distinct trajectory groups of CVD risk score: low–low, medium–medium, and high–high, i.e., no change at the level of risk during the time; however, in all trajectory groups, risk factors changed in favorable or unfavorable directions. As expected, the high-high and medium-medium risk groups had a significantly higher CVD incidence than the low-low risk group.

Clustering in our analysis indicated that overall low-risk individuals remained a low risk, and high-risk ones remained high risk during the time, so substantial change in risk categories was not the case, and the hazard of CVD remained stable. We observed a direct association between CVD risk score trajectory during the time and subsequent risk of CVD. It is well recognized that cardiovascular health metrics (i.e., smoking, diet, physical activity, body mass index, blood pressure, total cholesterol, and fasting glucose) are directly associated with incident CVD events [21]. In 2019, Wu et al. showed the association between changes in cardiovascular health scoring, assessed by the seven health metrics as mentioned above, and CVD events independent of baseline health status [22]. For instance, compared to individuals with a constantly worst cardiovascular health status, those who had the best overall cardiovascular health status over four years had a 79% lower risk of CVD incidence [22]. Still, in another study by von sloten et al., investigators found no relationship between the direction of change in the category of a composite metric of cardiovascular health and CVD risk [23]. For example, they showed that while the increase from a moderate- to a high-risk category of cardiovascular health resulted in a significant protective hazard ratio (HR < 1), a decrease from a high- to a low-risk category was also associated with a significant hazard ratio lower than one [23].

We revealed that the increased CVD risk score in the high-high and medium-medium trajectory groups became stable after keeping the age constant at baseline. The stable status of CVD risk score during time regardless of age may be explained by the steady trend of risk factors in trajectories. However, we showed that individuals' overall risk score is not in the same direction as their different risk factors are. In all trajectory groups, unfavorable increasing in fasting glucose, but favorable raising in HDL and decreasing smoking and total cholesterol happened over time.

Although exposure to multiple risk factors occurs throughout life and during aging, age per se is an independent and inevitable CVD risk factor [24]. This study highlights the potential impact of age on CVD during middle and later life. However, it is well recognized that the effect of risk factors on CVD events declines during aging [25]; this reduction may happen because low-risk individuals survive during the time. It is known that CVD risk factors only explain around 50% of the risk for CVD events, and the effect of age alone is substantial [26]. Compatible with our study, Bress et al. indicated that aging has the most significant influence on risk assessment scoring [27]. They reported that 60% of the development of high 10-year predicted CVD risk is attributable to aging [27]. In comparison, increased systolic blood pressure and incident diabetes mellitus accounted for 33% and 13% of the risk, respectively [27]. Increasing age raises CVD risk directly and indirectly by worsening risk factors such as lipids and blood pressure [28–30]; however, much of the risk is mediated by vascular dysfunction, including macro and microvascular endothelial dysfunction [28].

As a strength, our study is a large population-based longitudinal cohort study with repeated measurements of CVD risk factors over a substantial follow-up period. Furthermore, we used the Pooled Risk Equation recommended by ACC/AHA that is widely used in clinical practice and has the advantage of integrating several risk factors; this risk equation has been validated in the TLGS before [16]. Assessing the CVD risk score trajectories over ten years may provide insights into the association between long-term cardiovascular risk scores and subsequent CVD events.

Our study had some limitations that need to be mentioned. Foremost is that the TLGS only included urban adults in Tehran, which might reduce the generalizability of the results to other populations, mainly rural individuals. Second, we used repeated CVD risk scores over the four examination cycles to ensure sufficient follow-up time for the prospective analyses, so more extended variation in the CVD risk pattern might be missed. Finally, it is essential to note that the trajectory patterns identified by the group-based trajectory models only represent systematic attempts to classify individuals based on the available data, so they should not necessarily be interpreted as intrinsic properties.

## Conclusions

In conclusion, we showed that the CVD risk categories (high, medium, and low) are somehow stable during the time, which results in high-high, medium-medium, and low-low risk trajectories. Although the risk trajectories were stable during the time, different risk factors varied differently in each trajectory. These findings emphasize the importance of attention to each risk factor separately and implementing preventive strategies that optimize CVD risk factors besides the CVD risk. Further studies should focus more on how multiple CVD risk factors trajectories can predict CVD events instead of CVD risk trajectories.

## Supplementary Information

**Additional file 1: Table S1. **Model selection results.

**Additional file 2: Figure S1. **CVD risk score trajectories up to the examination cycle four; circles display the observed values while dotted lines represent fitted trajectories. CVD risk score was modeled as a function of time.

**Additional file 3: Figure S2. **CVD risk score trajectories up to the examination cycle four; circles display the observed values while dotted lines represent fitted trajectories. CVD risk score was modeled as a function of time.

## Data Availability

The datasets used during the current study are available from the corresponding author on reasonable request.

## Abbreviations

CVD: Cardiovascular disease
TLGS: Tehran Lipid and Glucose Study
ACC: The American College of Cardiology
AHA: The American Heart Association
CI: Confidence Interval
HDL: High-density lipoprotein
AvePPj: The average posterior probability of assignment for each group j
OCC: The odds of correct classification
SBP: Systolic blood pressure
FBS: Fasting blood sugar
GEE: Generalized estimating equation
