# Supplementary material for: Trajectories of cardiovascular disease risk and their association with the incidence of cardiovascular events over 18 years of follow-up: The Tehran Lipid and Glucose study
Source: J Transl Med. 2021 Jul 16;19:309. doi: 10.1186/s12967-021-02984-2 (PMC8284005; doi:10.1186/s12967-021-02984-2)
Supplement: Supplementary file 1 — Additional file 1: Table S1. Model selection results. [file 12967_2021_2984_MOESM1_ESM.docx]

| **Table S1.** Model selection results. | | | | |
| --- | --- | --- | --- | --- |
| Number of groups | Polynomial order | BIC | % Participants per group | Average posterior probabilities |
| 2 | 2 2 | 20935.42 | 83.3/16.7 | 0.99/0.95 |
| **3** | **2 2 2** | **20567.26** | **76.2/19.4/4.4** | **0.98/0.94/0.97** |
| 4 | 2 2 2 2 | 21741.73 | 20.3/66.1/11.1/2.4 | 0.89/0.97/0.94/0.99 |
| 5 | 2 2 2 2 2 | 24355.96 | 27.0/51.1/13.7/6.5/1.8 | 0.90/0.96/0.93/0.95/0.98 |
| **3** | **0 1 1** | **20398.59** | **73.9/21.5/4.6** | **0.98/0.95/0.97** |
| Reported are: the number of trajectory groups considered, the polynomial form of the model, the Bayesian Information Criterion (BIC), the posterior classification of subjects in each class (%), the average of posterior probabilities (AvePP) in each group. The best fitting model is highlighted in bold characters. | | | | |
