# Supplementary material for: Trajectories of cardiovascular disease risk and their association with the incidence of cardiovascular events over 18 years of follow-up: The Tehran Lipid and Glucose study
Source: J Transl Med. 2021 Jul 16;19:309. doi: 10.1186/s12967-021-02984-2 (PMC8284005; doi:10.1186/s12967-021-02984-2)
Supplement: Supplementary file 3 — Additional file 3: Figure S2. CVD risk score trajectories up to the examination cycle four; circles display the observed values while dotted lines represent fitted trajectories. CVD risk score was modeled as a function of time. [file 12967_2021_2984_MOESM3_ESM.docx]

**Figure S2.** CVD risk score trajectories up to the examination cycle four; circles display the observed values while dotted lines represent fitted trajectories. CVD risk score was modeled as a function of time.
